# Overcoming the challenge of the last mile

**Published:** 2022-09-20

**Authors:** Hannah Faal, Andrew Bastawrous, Elmien Wolvaardt

**Affiliations:** Adjunct Professor of International Eye health: University of Calabar Teaching Hospital, Calabar, Nigeria.; Professor in Global Eye Health: London School of Hygiene and Tropical Medicine, London, UK and Founder & CEO: Peek Vision; Editor: *Community Eye Health Journal*, International Centre for Eye Health, London School of Hygiene & Tropical Medicine, London, UK.

**Planning relevant and workable approaches to reach and support people in the last mile is critical if we are to increase the demand for, and uptake of, eye health services.**

**Figure:**
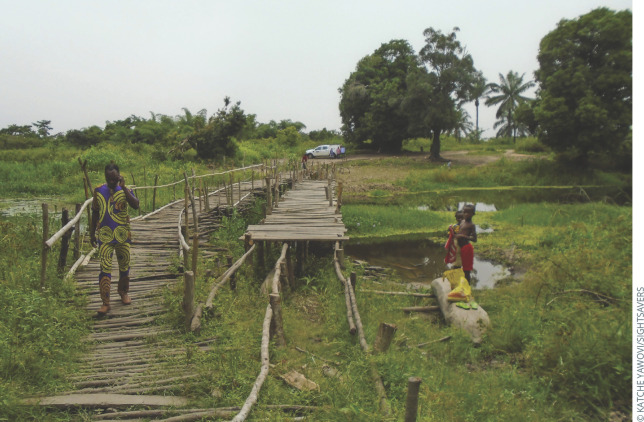
In rural areas, physical access is characterised by footpaths and broken or absent bridges. benin

Despite the significant achievements of the United Nations, Millennium Development Goals global project, millions of people are still being left behind, especially those living in poverty and those who are disadvantaged because of their sex, age, disability, or ethnicity. Three-quarters of these groups live in the ‘**last mile**’: i.e., remote or hard-to-reach areas that have insufficient provision of education, health care, water, and sanitation.,

The term ‘last mile’ was initially used by businesses to define which of their target customers were the last to receive specific products and services. More recently, development agencies have adopted the term to refer to populations who are the hardest to reach.

## Where is the last mile?

In **rural areas**, access to the last mile is characterised by footpaths, poor roads, and broken or absent bridges; in some seasons they may not be accessible at all. Transport is often limited to walking or cycling. For people living near rivers or in river deltas (such as the Sundarbans in India), access is made even more difficult due to floods, oil spillages, dependency on canoes or motor boats, and infrequent services.

“People living in the last mile are those who are not being reached by health care services; eye care services in particular.”

In **urban or peri-urban areas**, the last mile includes areas such as urban slums or ‘townships’ that are not connected to basic services such as water, sanitation, electricity, internet, cellphone access, and transport networks. In 2018, one billion people, or 24% of the world's urban population, were estimated to be living in urban slums.

## Who lives in the last mile?

People living in the last mile are those who are not being reached by health care services, including eye care services, and/or who cannot take the steps needed to reach out for the basic eye care or other health care services they need. They can include:

- **disadvantaged groups**, such as people living in extreme poverty, women, migrants, refugees, and internally displaced people (due to conflict, climate change, and/or natural disasters)
- **minority groups** (due to their ethnicity, religion, or sexual orientation) and indigenous communities
- **people with health or physical challenges**, such as older people who have chronic health conditions, people with disabilities (including intellectual disabilities), neurodiverse individuals (i.e., those with autism, dyslexia, dyspraxia, and/or attention deficit disorder), and people with mental health conditions (including depression, anxiety, and other conditions).

## Why is there a last mile?

Apart from physical distance, there are three factors that contribute to the existence of the last mile and keeps people trapped there: cultural or belief systems, values and prioritisation, and communication barriers.

**Cultural or belief systems.** Cultural or belief systems may stigmatise some groups or make them dependent on others for permission and/or the resources they need to seek health services: e.g., women, children, and widows in male-dominated societies.

**Values and prioritisation.** Political values often reflect society's values, and this can determine how and where resources are allocated. For example, health providers and governments may prioritise spending on specialist tertiary care (which benefits wealthier urban populations), while neglecting community and primary health care.

**Communication barriers.** These include physical barriers such as a lack of cellphone access or radio coverage. In addition, an absence of translated, localised, and culturally sensitive communication and health information strategies means that communities are not aware how important eye care services, such as glaucoma or diabetes screening, are for preventing visual impairment and blindness. As a result, eye care may not be a top priority for communities in the last mile unless there is an emergency or trauma, or in the late stages of disease when vision is already affected and cannot be restored.

## Why is reaching people in the last mile difficult?

**Data gaps and connectivity.** We know the least about those we are not reaching, which makes it harder to develop targeted, specific interventions. Communities in the last mile often do not have internet or cellphone connectivity, and therefore teleophthalmology and other real-time (or synchronous) data sharing activities – which optimise service delivery – may not be possible.

**Distance.** People in the last mile, by definition, are furthest from services, which presents a range of barriers. In urban slums, the geographical distance to service points may be relatively low, but the environmental degradation and poverty level may be so high that access to services remains impossible for the population.

**Cost.** The cost to overcome these barriers, for either patient or provider, may be too high. On the provider side, this may be because the cost of reaching the last mile was not included in national health or eye care plans, and so are not budgeted for.

## What is the implication for population eye health of poor last mile delivery?

People in the last mile are already members of the most disadvantaged group in society. It is staggering to realise that they are also the ones most likely not to receive the care they need – and will therefore remain visually impaired, in pain, or unsupported.

This equity gap will continue to grow unless the last mile is addressed. The sooner the better, since – as populations grow – the number of people in the last mile will also increase, making it even harder to reach them in an affordable way.

**Figure:**
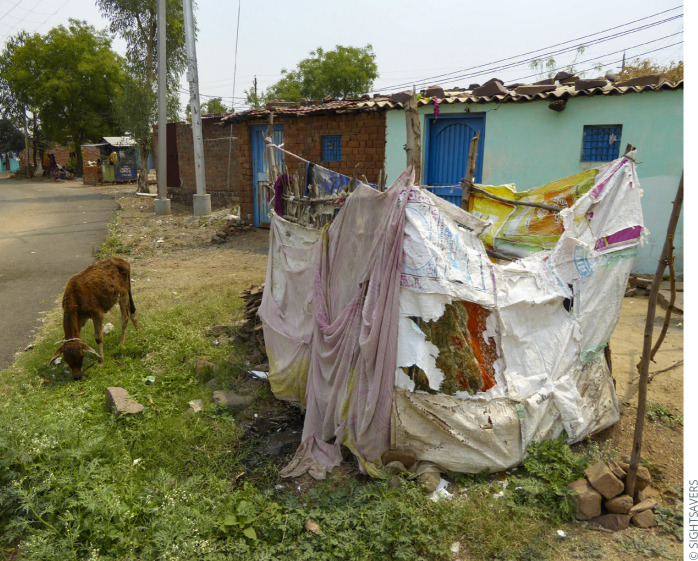
Over 1 billion of the world's population is estimated to live in urban slums, where access to eye care is also limited. india

## How to reach people in the last mile: practical steps

Establishing a primary eye health care service (see *Community Eye Health Journal* No. 113: Primary eye health care), as close to last mile communities as possible, is an important first step. The primary health care service not only functions as the base for developing outreach services to reach the last mile community, but also links people to the secondary and tertiary eye care services they may need.

Although every country and situation is different, the steps below can help with planning relevant and workable approaches to reaching people in the last mile and supporting them to increase the demand for, and uptake of, services.

### 1. Situation analysis

#### a. Define the population

Agree with governments or programme leaders on the criteria for defining last mile populations and/or locations (rural and urban).

#### b. Estimate the population size

Estimate the number of people living in the last mile (as defined) or use data from other public health programmes, e.g., polio immunisation or other population-wide health campaigns.

#### c. Estimate the need

Find out what proportion of the population in the last mile needs eye care services. For example, in our recent issue on primary eye health care, authors Clare Gilbert and Mapa Prabhath Piyasena estimated that 27% of community members in Asia, 20% of community members in Africa, and 17% of community members in Latin America needed preventive, curative, and rehabilitative eye care services.^4^ These estimates are very general, and will differ significantly between countries and among different groups in the same country. If available, use data from Rapid Assessment of Avoidable Blindness (RAAB) surveys, which produce detailed estimates of eye care need. Data from over 300 RAAB surveys worldwide are available, free of charge, at **www.raab.world**.

Using the known population size in the defined last mile area, and the estimated proportion of people who need care, you can work out the **number of people estimated to need care in the last mile**. This is the **denominator** against which to measure current access and any future progress.

#### d. Current access and gaps

If possible, find out how many people in the last mile population have had contact with eye care over the past year. Comparing this to the number of people estimated to need care gives an idea of the number of people who are not being reached at all.

However, simply reaching people is not enough. Referral and follow-up systems need to work well too. For example, try to find out: of those who were reached, what proportion received help such as near vision spectacles or eye drops immediately, and what proportion was referred? Of those who were referred, how many attended, and how many did not? What were the factors responsible?

Just as important is understanding the quality of the clinical care provided, and people's experience of the eye service. Poor outcomes and bad experiences (e.g., long waiting times or being treated as second-class citizens) will harm the reputation of the service and reduce demand and uptake.

#### e. Assessment of strengths: what is available in the last mile?

**The community**. Assess the strengths of the last mile community: to what extent are they able to (and do they already) participate in eye care that is offered, and take responsibility for their own health? Are they self-organising – i.e., are there existing structures and organisations in the community that can collaborate with eye care providers to improve access?

**Community systems: health and** **non-health.** Identify formal, informal, and traditional systems and services, and categorise them into helpful/useful and harmful (e.g., traditional practices such as couching).

### 2. Plan intervention strategies

Using the data, focus interventions on who is not being found and who is not attending referral appointments. How can services be adapted to better reach the community? How can people be empowered with the knowledge that they need eye care? How can they be supported to find and make use of the eye care services they need?

Some suggested approaches (in discussion and agreement with the population) are:

- **Shortening the last mile** using technology, mHealth, and telehealth; reducing travel by provider and population, and through specially designed last mile programmes.
- **Addressing cost barriers** through novel payment strategies and insurance schemes, and/or preferential budgets by government
- **Improving the quality of services**, both clinical and non-clinical, by developing special strategies to win trust and confidence in order to improve the demand for, and uptake of, services
- **Building on the strengths of the population in the last mile.**

Cost is an important factor and enough budget must be set aside to build trust and support long-term engagement with communities in the last mile, in order to ensure that communities are involved in a project from inception through to the monitoring and evaluation of services.

### 3. Evaluate, reflect, and make improvements

Continually monitor progress against the denominator (the number of people in the last mile who need eye care) and make changes where needed, in partnership with the community.

“Assess the strengths of the last mile community: to what extent are they able to (and do they already) participate in eye care that is offered, and take responsibility for their own health?”

**Figure:**
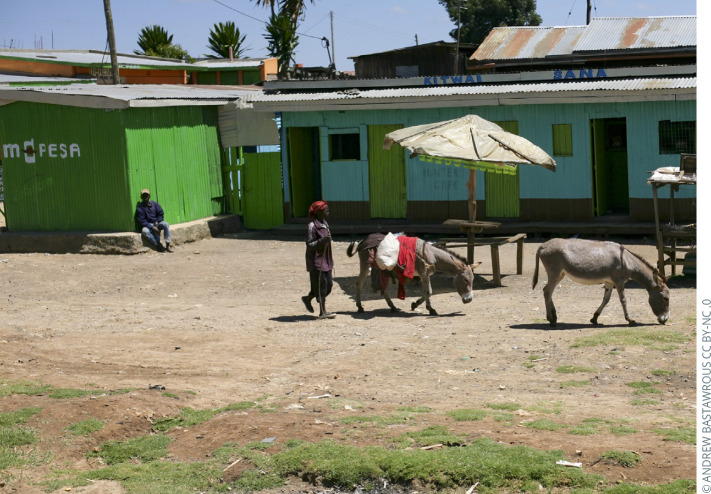
Novel strategies such as cellphone payments can support access to eye care in the last mile. kenya

### 4. Advocate for government investment

Alongside our efforts in eye care, we must also highlight the plight of people in the last mile and advocate for long-term government investment in the provision of basic services in last mile areas. As mentioned earlier, including the last mile in national health care plans is an important step in ensuring buy-in from the highest levels of government, including providing budgetary support. Reaching the last mile is also part of the United Nations target of universal health coverage, which includes minimising the geographical distance between populations and services, and providing essential services of high quality at an affordable cost, so that service recipients are not pushed into poverty or financial hardship. Governments worldwide adopted this target in 2015 and reaffirmed their commitment in 2019, and can therefore be held to account.^5^
